# Noninvasive continuous hemodynamic monitoring

**DOI:** 10.1007/s10877-012-9375-8

**Published:** 2012-06-14

**Authors:** Jasper Truijen, Johannes J. van Lieshout, Wilbert A. Wesselink, Berend E. Westerhof

**Affiliations:** 1 Laboratory for Clinical Cardiovascular Physiology, AMC Heart Failure Research Center, Amsterdam, The Netherlands; 2 Acute Admissions Unit, Department of Internal Medicine, Academic Medical Center, University of Amsterdam, Amsterdam, The Netherlands; 3 School of Biomedical Sciences, University of Nottingham Medical School, Queen’s Medical Centre, Nottingham, UK; 4 Clinical Team, BMEYE BV, Centerpoint 1, 4th floor, Hoogoorddreef 60, 1101 BE Amsterdam, The Netherlands

**Keywords:** Blood pressure, Cardiac output, Finapres methodology, Nexfin, CO-trek, Pulse contour analysis

## Abstract

Monitoring of continuous blood pressure and cardiac output is important to prevent hypoperfusion and to guide fluid administration, but only few patients receive such monitoring due to the invasive nature of most of the methods presently available. Noninvasive blood pressure can be determined continuously using finger cuff technology and cardiac output is easily obtained using a pulse contour method. In this way completely noninvasive continuous blood pressure and cardiac output are available for clinical use in all patients that would otherwise not be monitored. Developments and state of art in hemodynamic monitoring are reviewed here, with a focus on noninvasive continuous hemodynamic monitoring form the finger.

## Introduction

The primary evaluation of the hemodynamic condition is done by assessing heart rate (HR) and mean blood pressure (BP) as a surrogate of tissue perfusion. When these parameters change rapidly, a single measurement conveys insufficient information, making continuous measurement desirable [1]. For continuous measurement of BP, cannulation of an artery is the primary approach. However, noninvasive and continuous monitoring of BP has several advantages, particularly if intra-arterial measurement of BP is not warranted while intermittent measurements do not have the required time resolution [2]. Finger cuff technology can provide such continuous and noninvasive monitoring of BP and other hemodynamics parameters.

Although giving vital information, BP and HR due to their regulated nature frequently do not respond to substantial changes in intravascular volume, e.g. fluid administration or blood loss. Age and pre-existing cardiovascular morbidity complicate interpretation of these parameters further [3–9]. In supine adults hypotension and tachycardia are frequently absent even after blood loss of more than 1 l [3–8, 10, 11]. Therefore, fluid administration to optimize cardiac preload guided by BP is not straightforward. In contrast, cardiac output (CO) and especially cardiac stroke volume (SV) are sensitive to deviations in preload [7, 8]. Also, when arterial pressure is being restored by administering sympathomimetic drugs, it is at the expense of regional flow possibly including that to the brain [12]. Moreover, there is growing evidence that a patient’s cumulative fluid balance as well as strategy to guide fluid administration have an impact on patient morbidity and hospital stay. This has stimulated the development of methods that immediately detect changes in cardiac preload and output. With techniques like trans-esophageal and thoracic echocardiography or Doppler [13, 14], arterial pulse contour analysis [15–18] and determination of CO by lithium kinetics [19], several alternatives to the traditional indicator dilution and pulmonary artery catheterization techniques [20–22] have become available. These alternatives facilitate continuous and even noninvasive evaluation of volume treatment of patients [23]. Many continuous CO monitoring devices are called “minimally invasive” since they use arterial access that is already present for monitoring of BP or blood gas analysis. Nonetheless, the use of these devices is restricted to patients having such access. Recently, several completely noninvasive devices measuring continuous BP or CO were introduced for clinical use. The Nexfin^®^ (BMEYE B.V. Amsterdam, the Netherlands) allows hemodynamic monitoring with both BP and CO continuously available in patients without an arterial line. Continuous BP is measured with a cuff around a finger and a pulse contour method calculates beat-to-beat CO. This review summarizes past and present developments in BP and CO measurement with a focus on continuous noninvasive finger cuff technology and its clinical applications.

## Overview of methods for measurement of blood pressure

The first quantitative measurements of blood pressure were performed in animals by Hales in 1733 [24, 25]. Early reports of intra-arterial pressure measurement in the human are from 1912, when Bleichröder [26] cannulated his own radial artery. It is unlikely that he recorded his BP although it would have been possible at that time: Frank developed accurate and fast manometers that could measure pulsatile pressure in 1903 [27]. Invasive measurement of BP was confined to the physiology labs for quite some time [28, 29]. However in the 1950s and 1960s, with the development of refined insertion techniques [30] and Teflon catheters it became standard clinical practice. High fidelity catheter-tip manometers, such as used to measure pressure gradients across a coronary stenosis, were introduced by Murgo and Millar in 1972 [31]. Table 1 gives an overview of BP methods.

**Table 1:** Methods for measurement of blood pressure and cardiac output

System	Method	Company	CO		BP	
Nexfin	Finger cuff technology/pulse contour analysis	BMEYE	+	___	+	___
Finometer	Finger cuff technology/pulse contour analysis	FMS	+	___	+	___
LIFEGARD^®^ ICG	Thoracic electrical bioimpedance	CAS Medical Systems, Inc.	+	___	+	…
BioZ Monitor	Impedance cardiography	CardioDynamics International Corporation	+	___	+	…
Cheetah reliant	“Bioreactance”	Cheetah Medical	+	___	+	…
Cardioscreen/Niccomo	Impedance cardiography and impedance plethysmography	Medis Medizinische Messtechnik GmbH	+	___	+	…
AESCULON	Electrical “velocimetry”	Osypka Medical GmbH	+	___	+	…
HIC-4000	Impedance cardiography	Microtronics Corp Bio Imp Tech, Inc.	+	___		
NICaS	Regional impedance	NImedical	+	___		
IQ2	3-dimensional impedance	Noninvasive Medical Technologies	+	___		
ICON	Electrical “velocimetry”	Osypka Medical GmbH	+	___		
PHYSIO FLOW	Thoracic electrical bioimpedance	Manatec biomedical	+	___		
AcQtrac	Thoracic impedance	Väsamed	+	___		
esCCO	Pulse wave transit time	Nihon Kohden	+	___		
TEBCO	Thoracic electrical bioimpedance	HEMO SAPIENS INC.	+	___		
NCCOM 3	Impedance cardiography	Bomed Medical Manufacturing Ltd	+	___		
RheoCardioMonitor	Impedance cardiography	Rheo-Graphic PTE	+	___		
HemoSonic™ 100	transesophageal Doppler	Arrow Critical Care Products	+	___		
ECOM	Endotracheal bioimpedance	ConMed Corporation	+	___		
CardioQ-ODM™	Oesophageal Doppler	Deltex	+	___		
TECO	Transesophageal Doppler	Medicina	+	___		
ODM II	Transesophageal Doppler	Abbott	+	___		
HDI/PulseWave™ CR-2000	Pressure waveform analysis	Hypertension Diagnostics, Inc	+	_ _	+	_ _
USCOM 1A	Transthoracic Doppler	Uscom	+	_ _		
NICO	Rebreathing Fick	Philips Respironics	+	…		
Innocor	Rebreathing Fick	Innovision A/S	+	…		
Vigileo/FloTrac	Pulse contour analysis	Edwards Lifesciences	–	___	–	___
LiDCOplus PulseCO	Transpulmonary lithium dilution/pulse contour analysis	LiDCO Ltd	–	___	–	___
PiCCO2	Transpulmonary thermodilution/pulse contour analysis	PULSION Medical Systems AG	–	___	–	___
MOSTCARE PRAM	Pulse contour analysis	Vytech	–	___	–	___
Vigilance	Pulmonary artery catheter thermodilution	Edwards Lifesciences	–	…	–	___
DDG	Dye-densitogram analyzer	Nihon Kohden	–	…		
Truccom	Pulmonary artery catheter thermodilution	Omega Critical Care	–	…		
COstatus	Ultrasound dilution	Transonic Systems Inc.	–		+	…
CNAP Monitor 500	Finger cuff technology	CNSystems Medizintechnik AG			+	___
SphygmoCor^®^ CPV System	Applanation tonometry	AtCor Medical			+	_ _
TL-200 T-LINE	Applanation tonometry	Tensys Medical, Inc.			+	_ _

+ noninvasive, – invasive, ___ continuous, _ _ semi-continuous, … intermittent

Practical noninvasive (intermittent) BP measurement became possible when Riva-Rocci presented his air-inflatable arm cuff connected to a manometer in 1896 [32, 33]. By deflating the cuff and feeling for the pulse, systolic BP could be determined. In 1905 Korotkoff [34, 35] advanced the technique further with the auscultatory method making it possible to determine diastolic pressure as well. In 1903 Cushing recommended BP monitoring using the Riva-Rocci sphygmomanometer for patients under general anesthesia [36]. Nowadays, automated assessment of BP with oscillometric devices is commonly used. These devices determine BP by analyzing the oscillations measured in the cuff-pressure. The pressure in the cuff is first brought above systolic pressure and then deflated to below diastolic pressure. Oscillations are largest when cuff pressure equals mean arterial pressure. Proprietary algorithms determine systolic and diastolic values from the oscillations. Oscillometers may be inaccurate [37], and provided values that are frequently lower than direct BP measurements in critically ill patients, [38, 39] whereas detection of large BP changes is unreliable [40]. Due to its intermittent nature hyper- and hypotensive periods may be missed [2].

“Semi-continuous noninvasive methods” based on radial arterial tonometry require an additional arm cuff to calibrate arterial pressure [41–43]. The use of these devices may become problematic under conditions with significant patient motion or surgical manipulation of the limbs [43, 44]. However, tonometry devices have contributed greatly to the knowledge of the relation between the pressure wave shape and cardiovascular function [45, 46].

## Noninvasive continuous measurement of blood pressure

Continuous noninvasive measurement of BP is possible using finger cuff technology. The first generation using this technology was introduced with the Finapres™ device developed by Wesseling et al. [47] in the early 1980s. This technology is based on the volume-clamp method invented by the Czech physiologist Jan Peňáz [47–49]. The diameter of a finger artery under a cuff is “clamped” i.e. kept at a constant diameter in the presence of the changes in arterial pressure during each heart beat. Changes in diameter are measured by means of an infrared photo-plethysmograph built into the finger cuff. The finger cuff keeps the diameter of the underlying arteries constant by dynamically applying a counter-pressure throughout the cardiac cycle. When, for instance, during systole an increase in arterial volume is detected by the plethysmograph, the cuff pressure is immediately increased by a rapid pressure servo-controller system to prevent the volume change [17]. An artery could be clamped at any volume between collapsed and fully extended, but in either case the vessel wall will bear part of the pressure. Only when the artery is kept at its “unloaded” volume, there is no tension in the wall and internal pressure equals external pressure. Defining the correct unloaded volume of a finger artery is not straightforward. Moreover, the unloaded volume of an artery has to be established regularly since it is a function of arterial wall smooth muscle stress and tone. At zero transmural pressure the artery is not collapsed but retains approximately 1/3 or 1/2 of its maximal volume. The unloaded volume is also close to the volume it would have at a mean pressure where the amplitude of the pulsations in the plethysmogram are largest. The unloaded volume can be established by “Physiocal^®^”, developed by Wesseling et al. Physiocal analyzes the curvature and sharpness of the plethysmogram during short periods of steady cuff pressure levels [49]. A set of criteria allows determining whether the volume is precisely at a level that holds the optimum between the slightly too much collapsed and slightly too much extended volumes. The analysis is automatically repeated regularly during measurement, to follow changing physiological states of the vasculature. Owing to Physiocal, calibrated recordings of the entire finger arterial pressure wave are obtained [17]. Generally, an interval between Physiocal calibrations of more than 30 beats is accepted as a criterion for a reliable measurement. Pressure values are typically available within approximately 1 min after starting the measurement. While the Finapres and its successors use Physiocal, the CNAP device (CNSystems Medizintechnik AG, Graz, Austria) uses ‘interlocking control loops’ for volume clamping called the VERIFI algorithm. However, frequent calibration with an upper arm cuff is still needed [50].

**Fig. 1:**
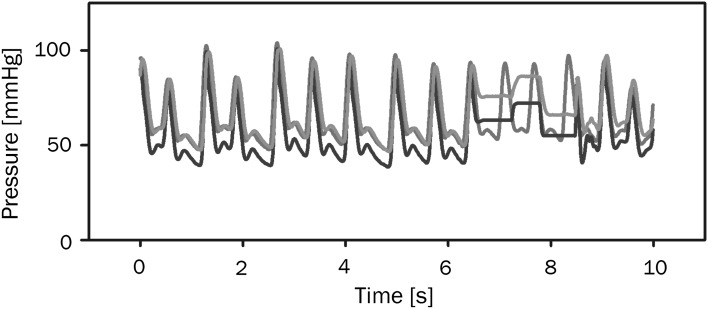
The differences in pressure levels and wave shape in the radial (*red*) and finger (*blue*) arteries. A physiological model can reconstruct the brachial artery pressure (*green*) from the finger arterial pressure. The staircase is the result of an automatic calibration. (Color figure online)

The Finapres device showed the arterial pressure as measured at the finger. However, since the brachial site is the clinical standard for noninvasive BP measurement later devices such as the Finometer (FMS, Amsterdam, the Netherlands) and the Nexfin (BMEYE, Amsterdam, the Netherlands) show the brachial pressure reconstructed from the finger pressure. Reconstruction reverses the physiological waveform transformation that waves experience while travelling through the arterial system (see Fig. 1). The progressively narrowing arteries cause backwards reflection of the pressure waves, resulting in a more peaked waveform towards the periphery. Additionally, in the smaller arteries, the resistance starts to play a role and pressure levels become affected. The change in waveform from the central to the peripheral arteries is largely predictable [51–53]. The waveform transformation along the arm can be mathematically described and this description can be used to reverse the transformation [54, 55]. The pressure drop due to resistance to flow in the smaller arteries can be compensated by application of a level correction formula. This population based formula determines a pressure drop based on systolic and diastolic values [51]. The combination of these two methods reconstructs brachial artery pressures from finger arterial pressures. An alternative is used in the CNAP device that displays a finger pressure wave that is fitted to systolic and diastolic pressures from upper arm cuff measurements.

Both the Finometer and the Nexfin use a system that automatically corrects for hydrostatic differences in pressure when the hand is not at heart level. The “finger side” of the heart reference system is fixed next to the finger on which the cuff is applied and the “heart side” at right atrial level. The hydrostatic difference is measured and the recordings are continuously corrected to give BP at heart level. The CNAP device uses repeated upper arm cuff measurements to compensate for hydrostatic pressure differences.

## Validation of noninvasive continuous measurement of blood pressure

Tracking of changes in BP was already considered good in earlier devices using the finger cuff technology [56]. With the physiological waveform transformation absolute values reconstructed from finger arterial pressures are comparable with (non-) invasively measured brachial or radial pressures [57–59]. Measurements with the Nexfin are performed without the need for an external calibration whereas other devices using finger cuff technology such as the Finometer [17, 60] and the CNAP require extra measurements with an upper arm cuff [61]. A recent overview of noninvasive BP monitors and clinical validation studies focused specifically on the need for calibration by a separate method [62].

The BP measurement with the latest generation finger cuff technology device, the Nexfin, was validated against both invasive and noninvasive methods [59, 63, 64]. From a comparison against an auscultatory BP measurement (Riva-Rocci/Korotkoff) in 104 subjects it was concluded that Nexfin provides accurate measurement of BP with good within-subject precision [59]. Validation against invasive radial pressure was performed in fifty patients during coronary artery bypass grafting [64]. Within-patient analyses showed excellent correlations between the noninvasive and invasive pressures and good within subject precisions over wide ranges of pressure changes. Moreover, bias and precision, defined as group average and standard deviation of the differences, were within AAMI criteria [65]. No relation was found between the differences and mean arterial pressure or HR, indicating that the reconstruction methodology performs well in a wide range of hemodynamic states. It was concluded that noninvasively measured blood pressure could follow changes in pressure and provided values comparable to invasive monitoring.

The Nexfin has also been evaluated in an emergency care setting where the authors concluded that continuous BP and HR measured by the Nexfin device showed reasonable agreement when compared with the intermittent values obtained by automated ED equipment [66]. However, they also suggested that theoretically, noninvasive and continuous monitoring of the BP and HR might better reflect underlying hemodynamics than these same measurements obtained intermittently and, thus, could be important in patient management [66].

## Overview of methods for measurement of cardiac output

Various methods to measure CO are being used and can be characterized by their invasiveness or their ability to measure continuously. Table 1 gives an overview of CO methods. One of the first methods to determine flow was proposed by Fick [67], and uses the relation between the rate of uptake of oxygen in an organ and the difference of oxygen concentrations over that organ. Therefore, to measure CO (total flow in the body), arterial and mixed venous oxygen concentrations as well as oxygen consumption need to be sampled. Frank, also known for the Frank-Starling law of the heart, developed the 2-element Windkessel model to determine CO [68]. The two elements of this model are total arterial compliance and systemic vascular resistance. After total arterial compliance was estimated by a pulse wave velocity measurement, systemic vascular resistance could be determined from the diastolic decay of the pressure curve. Using Ohm’s law, CO could then be calculated by dividing mean arterial pressure by this resistance. This method can be seen as a very early pulse contour method, in which the shape of the pressure wave is analyzed to obtain CO.

Indicator dilution techniques use the Stewart-Hamilton [69] equation to describe the rate at which an indicator, injected into the blood stream, is diluted. CO is calculated from the quantity of injected indicator and the area under the Stewart-Hamilton curve measured downstream. The indicator usually is a dye or a thermal marker, and is injected into a vein and following passage through the heart subsequently sampled from an artery. For the thermodilution technique iced glucose is injected in the right atrium and the temperature downstream in the pulmonary artery is sampled with the Swan-Ganz catheter [70]. To obtain a reliable estimation with these techniques CO should remain constant for at least the duration of a single measurement. However, HR, SV and BP may change rapidly and this has resulted in the practice to perform several estimations of CO, and even sometimes in elimination of outliers from a series of consecutive thermodilution estimates, both indicating that the requirement of stability is usually not met.

Determination of CO with the thermodilution method is in general restricted to the critically ill patients or patients at high risk associated with an intervention or with serious comorbidities. The use of intermittent estimation of CO by thermodilution is decreasing [20–22] owing to the invasive nature of pulmonary artery catheterization. Besides the requirement to be less invasive, it may also be necessary to measure hemodynamic changes with short time intervals. Hereto, monitors need to provide a continuous measure of CO, usually based on analysis of the arterial BP curve (“pulse contour” methods).

In their publication of 1904 Erlanger and Hooker determined cardiac SV from characteristics of the arterial pressure pulse [71]. Pulse contour methods are based on solid physical principles, less solid physiological models, and involve substantial computations [72]. Until recently, pulse contour methods analyzed the arterial pulse wave from an intra-arterial catheter, initially in place for BP monitoring or sampling for blood gas analysis. Several minimally invasive techniques have become available that provide continuous CO measurement [73] operating either with or without an additional (invasive, intermittent) calibration. The LiDCO^®^ system applies a bolus indicator dilution method for CO measurement using lithium chloride as an indicator. Detection of the indicator in arterial blood through a lithium-sensitive electrode produces a lithium concentration–time curve [19]. The produced CO estimate is used to calibrate a pulse contour-derived SV. The PiCCO^®^ system also utilizes pulse contour analysis of intra-arterial BP for continuous CO monitoring, whereas transpulmonary thermodilution is used for calibration [74]. Although still invasive to some extent an advantage of these techniques over conventional thermodilution is that pulmonary artery catheterization is not required. The FloTrac/Vigileo™ system also uses an intra-arterial pressure waveform based pulse contour analysis [75–77], but without the need to calibrate.

In general, calibration may improve the accuracy of the absolute values, but is not essential as long as changes in CO are accurately tracked. Continuous tracking is necessary to assess the response to a relatively fast hemodynamic change, such as a fluid challenge or passive leg-raising. Responding to the challenge with a certain increase in CO indicates that the patient is fluid responsive and thus may benefit from receiving additional fluid [7, 9, 78]. A recent meta-analysis comprehensively reviews current minimally invasive CO techniques [79].

## Noninvasive continuous measurement of cardiac output

Noninvasive and (semi-)continuous tracking of changes in SV can be accomplished by thoracic electrical impedance [80–82], ultrasound [14, 83] and by pulse contour analysis [84–88]. Pitfalls of the first method include electrode placement, motion artifacts, validity of the applied equations, and calibration [89, 90]. Doppler ultrasound measurement of aortic blood velocity combined with echocardiographic estimation of the aortic root cross-sectional area yields SV, although semi-continuous at best. The application of the semi-invasive trans-esophageal approach is limited to anesthetized patients. For a transthoracic approach the Doppler probe has to be held over the root of the aorta requiring a skilled operator and constancy of probe angle to minimize bias [83]. Nonetheless, esophageal Doppler has been shown to give positive results in goal-directed therapy [91–93].

**Fig. 2:**
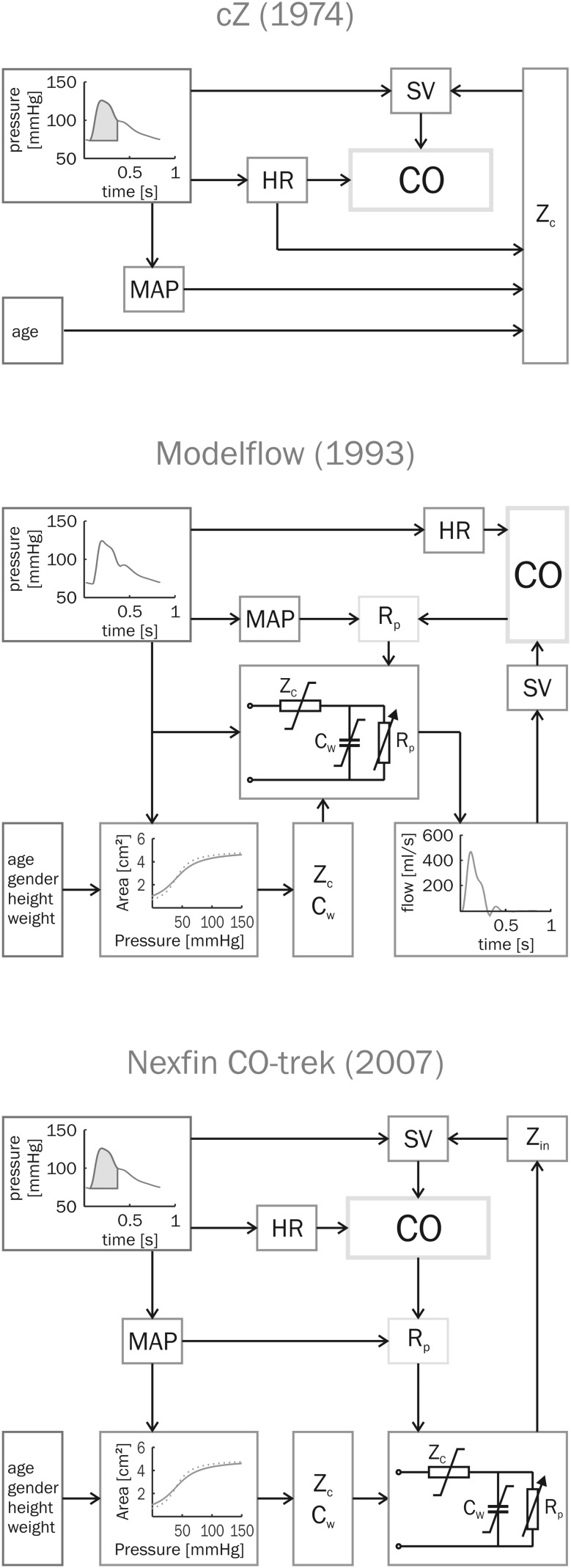
The cZ pulse contour method, Modelflow and Nexfin CO-trek. The corrected Z (“cZ”, *top*) method uses an impedance Z to calculate stroke volume (SV) [121]. The time-integral of the arterial pressure wave above diastolic pressure and between valve opening and closing (as determined by upstroke and incisura) is called the pulsatile systolic area (PSA, *hashed area* in the Figure) of the pressure. This PSA together with the characteristic impedance Z_c_, which is estimated from age and depending on mean arterial pressure (MAP) and heart rate (HR, the inverse of the heart period of the pressure wave), gives SV. Cardiac output (CO) is calculated by multiplying SV and HR. The method was developed for tracking of changes in CO and for correct absolute values a calibration of Z was necessary. In the Modelflow method (*middle*) the 3-element Windkessel was implemented. Z_c_ and Windkessel compliance C_w_ depend on age, gender, height and weight and are nonlinearly related to pressure as shown in the pressure—area relation [97]. Thus, patient data and arterial pressures are both needed to determine these Windkessel parameters [99]. The time varying R_p_ is the ratio of MAP and CO and iteratively determined. To calculate CO, Modelflow imposes arterial pressure on the Windkessel model. A flow curve is produced of which the time-integral gives SV. The Nexfin CO-trek method (*bottom*) divides the PSA by the input impedance of the Windkessel to instantaneously give SV. In contrast to Modelflow, there is no need for constructing a flow curve. Moreover while Modelflow was developed to be used on invasive pressures, Nexfin CO-trek was developed to work with noninvasive BP as measured with Nexfin [58].

In order to determine beat-to-beat SV and CO from noninvasive continuous BP from the finger a pulse contour method based on a physiological model of the circulation is used. In the early 1970s Wesseling developed the cZ pulse contour method making use of the systolic part of the pressure curve (pulsatile systolic area, PSA, see Fig. 2). This cZ method used a constant impedance Z to calculate the SV from the PSA. An empirical formula incorporating patient age, HR and mean arterial pressure was used to dynamically correct the impedance Z (hence the name cZ for corrected impedance) for changes in the hemodynamic status. Tracking of SV changes was reliable, but calibration of Z with a reference method was necessary for correct absolute values. Subsequent to the cZ method, Wesseling et al. developed a method using a 3-element Windkessel description of aortic input impedance. By adding a third element to Frank’s 2-element Windkessel, Westerhof et al. [94, 95] substantially improved the modeling of the pressure-flow relation [96]. The third element, the characteristic impedance Z_c_ represents the impedance that the ventricle encounters during ejection. Its value is determined by the interaction of the capacity of the proximal aorta to store volume (thus proximal compliance C) and the blood mass that needs to be accelerated (inertance L). Z_c_ is equal to the square root of L/C. Windkessel compliance C_w_ is equal to the sum of the compliances of all arteries, mainly the ascending and descending aorta, and it represents the ability to elastically store the stroke volume ejected by the heart. Peripheral resistance R_p_ equals the sum of all resistances of small arteries and arterioles, representing the resistance to the flow of blood. In Wesseling’s Modelfow method a Windkessel model is used in which Z_c_ and C_w_ are nonlinear functions of pressure, age, gender, height and weight [97] assuring better absolute CO values and tracking over a wider range of pressures [98]. Using pressure as input a flow curve could be calculated [18, 99, 100]. Subsequent integration of the flow curve yielded SV.

Nexfin CO-trek is a model implemented in the Nexfin that calculates beat-to-beat SV by dividing the area under the systolic part of the reconstructed brachial artery pressure curve by the aortic input impedance (Z_in_, Fig. 2). Z_in_ is determined from a 3-element Windkessel model [94, 96] with nonlinear descriptions for the parameters as proposed by Wesseling. Values for SV become instantaneously available with the onset of the BP measurement. Using the described physiological models for pressure reconstruction and input impedance, Nexfin CO-trek was internally evaluated on data including invasive and noninvasive finger arterial pressures together with thermodilution CO data obtained during cardiac surgery [98, 99], from healthy subjects experiencing progressive central hypovolemia induced by passive head-up tilt [15], and from critically-ill patients with arterial hypotension due to severe septic shock and treated with catecholamines [16]. Thermodilution CO in these trials was determined with quadruple respiratory phase controlled thermodilution estimates [101]. A specific objective for the development of Nexfin CO-trek was that noninvasive arterial pressure should be employed as input.

## Validation of noninvasive continuous measurement of cardiac output

Thoracic electrical impedance and ultrasound measurement of CO have been extensively described in the literature. Raaijmakers concluded that “impedance CO might be useful for trend analysis” [102]. Coats concluded that “Doppler methods are safe, fairly reproducible and reasonably accurate” [103]. It should be noted that, although ultrasound is often used to assess fluid responsiveness, the change in aortic diameter may give rise to substantial errors when not accounted for [104].

In a recent study both noninvasive finger arterial pressure and intra-arterial pressure input were used as input to two validate methods: Nexfin CO-trek and Modelflow. Awake post-coronary artery bypass surgery patients with a pulmonary artery thermodilution-based estimate of CO serving as a reference were included [58]. Measurements were done with patients in supine and sitting position. It was found that Nexfin CO-trek readings were comparable to thermodilution CO, with intra-arterial pressure as well as with noninvasive finger arterial pressure as input. The earlier Modelflow CO-method, developed to be used with invasively measured pressures, performed less well on noninvasive measured BP [58].

Sokolski et al. examined 25 ICU patients with advanced heart failure to compare CO measurements using the pulmonary artery catheter thermodilution method and using the Nexfin [105]. The reported bias and standard deviation were 0.1 and 0.4 l/min, respectively. These results are promising, especially considering the fact that the population included 13 patients with atrial fibrillation and 13 patients with decompensated heart failure. The authors therefore conclude that the Nexfin could be applied in clinical practice for patients with advanced HF.

Broch et al. concluded that “the Nexfin is a reliable method of measuring cardiac output during and after cardiac surgery” [106]. Compared with trans-cardiopulmonary thermodilution, the mean bias of Nexfin was −0.1 (95 % limits of agreement −0.6 to +0.5, percentage error 23 %) and −0.1 (−0.8 to +0.6, 26 %) l min^−1^ m^−2^, before and after cardiopulmonary bypass, respectively. These data show that Nexfin gives excellent results without calibration. Further, a good correlation between the two methods was found when passive leg-raise was performed, with R^2^ = 0.72, *p* < 0.001 before and R^2^ = 0.76, *p* < 0.001 after cardiopulmonary bypass.

Two recent studies further demonstrated the ability of tracking changes in CO. The first compared the changes in SV measured by Nexfin CO-trek with the echo Doppler aortic velocity–time integral as a measure of SV during the optimization of atrioventricular delay in cardiac resynchronization therapy and a good agreement was found [107]. The second study compared, during exercise, the CO of Nexfin CO-trek with inert gas rebreathing method for CO estimation [108]. A good correlation was found and values actually converged for large CO values [109].

## Clinical applications of noninvasive continuous hemodynamic monitoring

### Anesthesiology

Continuous, totally noninvasive monitoring is possible in groups currently (nearly) unmonitored. Examples include orthopedic surgery in the elderly, abdominal surgery and bariatric surgery. In obese patients upper arm cuffs for BP measurement often do not fit, and thigh cuffs are needed, or a brachial cuff is used on the forearm. While the arms and legs can increase significantly in circumference, fingers do get larger but usually not up to the degree that the finger cuff does not fit.

For surgical patients volume treatment corrects a perioperative volume deficit and attenuates negative influences on the central blood volume (corresponding to the diastolic volume of the heart) caused by, e.g. hemorrhage, repositioning of the patient, anesthesia and ventilation. Interpretation of the heart rate (HR) and arterial pressure responses to a reduced central blood volume is complex. Cardiovascular variables are regulated and affected by influences other than central blood volume, including surgical stress and anesthesia [10]. This makes it unlikely that accurate volume treatment can be based on HR and BP alone. Considering the important contribution of a subnormal central blood volume in circulatory shock, a definition of normovolemia may be derived from individualized goal-directed volume therapy, not only to the patient in shock but also to patients in the perioperative period [8]. Cerebral blood flow and oxygenation become affected with a blood loss corresponding to 30 % of the central blood volume [110] or a blood loss of 1.0–1.5 l [9]. It is becoming clear that monitoring of the circulation allows for intervention well before cerebral blood flow and oxygenation become affected [111]. Fluid therapy guided by cardiac output has been demonstrated to improve perioperative outcome and reduce complications and the length of hospital stay [91, 92, 112]. This goal-directed volume treatment is guided by various techniques that determine cardiac output [92, 93]. The availability of noninvasive and continuous monitoring of SV or CO enables individualization of fluid treatment from fixed-volume to goal-directed volume therapy in a wide range of patients.

### Emergency care

Noninvasiveness and ability for quick assessment allows the characterization of hemodynamic profiles of patients in the Emergency Department and following of possible changes. It was demonstrated by Nowak et al. [113] that emergency physicians, when asked whether the CO of their patients was low, normal or high, were right only half of the time. Nonetheless decisions in acutely ill patients are based on such assumptions of the underlying hemodynamic profile [113] with potentially important clinical ramifications.

### Cardiology

In cardiac resynchronization therapy, the atrioventricular delay or the inter-ventricular delay can be chosen to optimize SV. In a study comparing noninvasive pulse contour SV with echo Doppler aortic velocity–time integral while optimizing the atrioventricular delay, a good agreement was found and the authors concluded that Nexfin is a promising tool in individual optimization [107].

During invasive electrophysiology procedures, it is common practice to use an intra-arterial line to monitor BP in critical situations of hypotension caused by tachyarrhythmias or by intermittent incremental ventricular temporary pacing till to the maximally tolerated systolic BP fall. During such procedures Nexfin recorded reliable BP waveforms notwithstanding the presence of tachyarrhythmia [114]. The authors stated that continuous noninvasive BP monitoring is feasible in the interventional electrophysiology laboratory and may replace intra-arterial BP in that setting.

Noninvasive BP with finger cuff technology has been used for a long time in the diagnosis and management of syncope [115] and is used in tilt table testing and other autonomic function testing [116]. Nexfin enables continuous cardiovascular evaluation of patients presenting with unexplained syncopal attacks and thus considerably contributed to diagnostic efficacy and accuracy [117]. Also in this field CO is receiving increasing attention [118].

## Conclusion and perspective

As indicated by De Waal et al. [119] the ideal CO monitor should be: “reliable, continuous, noninvasive, operator-independent, cost-effective, and should have a fast response time (beat-to-beat).” Noninvasive and continuous determination of CO with the Nexfin is comparable to thermodilution CO, is continuous, truly noninvasive and operator independent. Generally within a minute after start-up, beat-to-beat data on BP, HR, SV and CO become simultaneously available. The only point of contact with the patient is the cuff around a finger. CO monitoring is not routine practice yet due to its often invasive and intermittent nature. Nonetheless, fluid therapy guided by CO has been shown to improve perioperative outcome and to reduce complications as well as length of hospital stay [112, 120]. Noninvasive continuous CO techniques make routine monitoring of CO readily available facilitating easier assessment of fluid-responsiveness and a further application of goal directed fluid therapy.
